# Comparative genomics of *Metarhizium brunneum* strains V275 and ARSEF 4556: unraveling intraspecies diversity

**DOI:** 10.1093/g3journal/jkae190

**Published:** 2024-08-30

**Authors:** Alexandra M Kortsinoglou, Martyn J Wood, Antonis I Myridakis, Marios Andrikopoulos, Andreas Roussis, Dan Eastwood, Tariq Butt, Vassili N Kouvelis

**Affiliations:** Section of Genetics and Biotechnology, Department of Biology, National and Kapodistrian University of Athens, 15771 Athens, Greece; Department of Biosciences, Faculty of Science and Engineering, Swansea University, Singleton Park, SA2 8PP, Swansea, UK; Section of Genetics and Biotechnology, Department of Biology, National and Kapodistrian University of Athens, 15771 Athens, Greece; Section of Genetics and Biotechnology, Department of Biology, National and Kapodistrian University of Athens, 15771 Athens, Greece; Section of Botany, Department of Biology, National and Kapodistrian University of Athens, 15784 Athens, Greece; Department of Biosciences, Faculty of Science and Engineering, Swansea University, Singleton Park, SA2 8PP, Swansea, UK; Department of Biosciences, Faculty of Science and Engineering, Swansea University, Singleton Park, SA2 8PP, Swansea, UK; Section of Genetics and Biotechnology, Department of Biology, National and Kapodistrian University of Athens, 15771 Athens, Greece

**Keywords:** endophytic entomopathogenic fungi, *Metarhizium brunneum*, whole genome sequencing, biosynthetic gene clusters (BGCs), CAZymes, transposable elements (TEs)

## Abstract

Entomopathogenic fungi belonging to the Order Hypocreales are renowned for their ability to infect and kill insect hosts, while their endophytic mode of life and the beneficial rhizosphere effects on plant hosts have only been recently recognized. Understanding the molecular mechanisms underlying their different lifestyles could optimize their potential as both biocontrol and biofertilizer agents, as well as the wider appreciation of niche plasticity in fungal ecology. This study describes the comprehensive whole genome sequencing and analysis of one of the most effective entomopathogenic and endophytic EPF strains, *Metarhizium brunneum* V275 (commercially known as Lalguard Met52), achieved through Nanopore and Illumina reads. Comparative genomics for exploring intraspecies variability and analyses of key gene sets were conducted with a second effective EPF strain, *M. brunneum* ARSEF 4556. The search for strain- or species-specific genes was extended to *M. brunneum* strain ARSEF 3297 and other species of genus *Metarhizium*, to identify molecular mechanisms and putative key genome adaptations associated with mode of life differences. Genome size differed significantly, with *M. brunneum* V275 having the largest genome amongst *M. brunneum* strains sequenced to date. Genome analyses revealed an abundance of plant-degrading enzymes, plant colonization-associated genes, and intriguing intraspecies variations regarding their predicted secondary metabolic compounds and the number and localization of Transposable Elements. The potential significance of the differences found between closely related endophytic and entomopathogenic fungi, regarding plant growth-promoting and entomopathogenic abilities, are discussed, enhancing our understanding of their diverse functionalities and putative applications in agriculture and ecology.

## Introduction

Numerous fungal families contain specialized species that can infect and kill a broad range of invertebrate hosts (Islam *et al*. 2021), with over 750 entomopathogenic fungal (EPF) species of 85 genera described to date (Paschapur *et al*. 2021). As such, there has been a steady increase in interest, discovery, research, and development of these species as biological control products. They have shown high utility within pest and vector control markets (Paschapur *et al*. 2021) as practical replacements for traditional chemical insecticides (Butt *et al*. 2016; Dauda and Maina 2018). Of the currently described EPF species, the genus *Metarhizium* (Sordariomycetes: Hypocreales: Clavicipitaceae) has been a leading participant in global biological control products (Altimira *et al*. 2022), e.g. *Metarhizium anisopliae* FI-985 (Green Guard) and *M. brunneum* V275 (Lalguard or Met52). In particular, strain V275 is among the most widely applied and efficient EPF commercial products (Long and Hunter 2005; Quesada-Morraga *et al*. 2006; Asan *et al*. 2017), while strain *M. brunneum* ARSEF 4556 has a similar high potential for exploitation (Wood *et al*. 2022; Alkhaibari *et al*. 2023).

*Metarhizium* spp. are ubiquitous in soil, exhibiting entomopathogenic life phases following direct contact between an arthropod host and fungal conidia (Butt *et al*. 2016; Mannino *et al*. 2019; St Leger and Wang 2020). The genus includes both early diverging species, which typically have a narrower insect host range and are termed as specialists, and more recently diverged species, such as *M. brunneum*, that tend toward generalists in host range (Hu *et al*. 2014). Several of the latter species, however, have recently been shown to exhibit alternate life modes, associating with a wide range of cultivated and wild plant species as beneficial endophytes, rhizosphere colonizers, and saprophytes (Meyling and Eilenberg 2007; Neiro *et al*. 2010; Garcia *et al*. 2011; Lopez and Sword 2015; Clifton *et al*. 2018; Dash *et al*. 2018). This relationship appears to be mutually beneficial; the plant offers refuge, nutrition, and host–insect access (Pineda *et al*. 2017; Mantzoukas and Eliopoulos 2020), while the fungus can confer a range of benefits, including plant growth promotion, plant-pathogen antagonism (Sasan and Bidochka 2013; Keyser et al. 2016; Jaber and Ownley 2018) and deterrence of invertebrate plant pests (Canassa *et al*. 2020; Francis *et al*. 2022). While the infection process in invertebrates is well described and understood (Butt *et al*. 2016; Hong *et al*. 2024), the mechanisms employed by EPF when colonizing plant tissues and transition between nutritional modes are poorly described (Fadiji and Babalola 2020; Ghosh *et al*. 2020; Mantzoukas and Eliopoulos 2020). Endophytic behavior and capacity differ between fungal strains, underscoring the complex nature of species interactions and a potential diversity in synchronous processes driving endophytic colonization (Fadiji and Babalola 2020; Mantzoukas and Eliopoulos 2020).

The increasing availability of whole genome sequences of *Metarhizium* strains (25 to date) ultimately led to the discovery of a range of fungal secondary metabolites including insecticides, immunosuppressants, and antimicrobials (Wang *et al*. 2012; Hu *et al*. 2014; Sbaraini *et al*. 2016). These metabolites are implicated in a range of important adaptational functions and generally arise from pathways that are not directly related to growth or reproduction but are key determinants of interactions and stress responses within their environment (Roberts and St Leger 2004; Keller 2015). The genes required for the biosynthesis of these compounds are usually organized as biosynthetic gene clusters (BGCs) and include the core genes encoding the synthesis of the conserved structural motif of each compound. Further tailoring enzymes, transcription factors, and transporters regulate the synthesis, modification, and targeting of the produced compound or its detoxification (Keller 2015). In addition, comparative analyses of gene complement of Carbohydrate Αctive Enzymes (CAZymes), pathogen-host interaction (PHI) genes, and other genome structural components, such as transposable elements (TEs), help determine the enzymatic machinery and other evolutionarily conserved factors that have shaped and continue to facilitate both entomopathogenic and endophytic modes of life in EPF (De Melo *et al*. 2013; Altimira *et al*. 2022).

In this study, a high-quality genome of the model EPF fungus *M. brunneum* strain V275 is reported. In addition, intraspecies comparative analyses with another high-quality genome of *M. brunneum* strain ARSEF 4556 (Saud *et al*. 2021) are performed. Both EPF strains present endophytic activity with differentiation observed related to their efficacy in employing their different modes of life. Therefore, in this study, their genomes’ comparative analyses aim to explore the molecular mechanisms underlying the unique endophytic and entomopathogenic characteristics of each of the two *M. brunneum* strains. An emphasis will be given on unique BGCs and CAZymes as well as gene singletons, TEs, and other genetic elements. By comparing gene sets of these strains with other *Metarhizium* species, our objective is to identify key factors contributing to their efficacy as both endophytes and entomopathogens. Through comparative bioinformatic analyses, we seek to elucidate variability in gene and protein content, and metabolic pathways associated with these attributes. The findings of this research could have significant implications for understanding and enhancing the effectiveness of *Metarhizium* strains in agricultural and biotechnological applications.

## Materials and methods

### DNA extraction and sequencing

*M. brunneum* strain V275 (ARSEF F52) strain was acquired from the ARS collection of entomopathogenic fungal cultures (ARSEF), and it was cultivated in potato-dextrose-agar medium for 7 days in the dark at 25 ± 1^°^C, before extraction. DNA was extracted with HigherPurity Plant DNA Purification Kit (Canvax Biotech, Spain) using 100 mg of fungal material, according to the manufacturer protocol. DNA quality was assessed by electrophoresis (0.8% agarose gel), as well as Nanodrop measurements and quantified using a Qubit fluorometer.

Illumina sequencing was performed using the INVIEW Resequencing package (Eurofins Genomics, Germany) and 2 × 150 bp paired-end reads were obtained. Sequencing quality was assessed using FASTQC (v0.11.9) (Babraham Bioinformatics Cambridge, UK) and adapters were removed using Trimmomatic (v0.39) (Bolger *et al*. 2014). To estimate genome size and other genome statistics, computational estimation was performed using k-mer occurrence distribution. For this aim, Jellyfish (v2.3.0) (Marçais and Kingsford 2011) was used with default parameters (*k* = 21) and Illumina PE reads as input, while the produced histogram was visualized using GenomeScope (Vurture *et al*. 2017).

Sequencing with Nanopore was performed using the MinION (MIN-101B) Oxford Nanopore Starter Pack (Oxford Nanopore Technologies, UK) device and R.10.4.1 flow cell (FLO-MIN114, Kit 14 chemistry). The sequencing library was prepared using the ligation sequencing kit SQK-LSK112 (Oxford Nanopore Technologies), following the manufacturer's protocol. Sequencing was carried out using MinKNOW (v4.2.5) software. Basecalling was performed locally with Guppy Software (v6.4.6) (Oxford Nanopore Technologies), using the super accurate (sup) model and the following parameters: –config dna_r10.4.1_e8.2_400bps_sup.cfg –bam_out cuda:0:95% –detect_adapter –trim_adapters –do_read_splitting –detect_mid_strand_adapter.

### Genome assembly and statistics

The nanoporebasecalled reads that remained after quality control (characterized as “pass’’) were used to remove adapters using Porechop (v0.2.4) (Wick *et al*. 2017), with 20.3 Gb of sequenced reads retained (out of 21.5 Gb). Filtlong (v2.0.1) (Wick 2017) was used to assess the quality and length of Nanopore reads by using Illumina reads as an external reference and according to the results, 90% of reads were retained. Illumina reads were then used to perform error correction of Nanopore long reads with FMLRC2 (Mak *et al*. 2023), using msbwt2 (Holt and McMillan 2014) to build the burrows wheeler transform. The output of corrected reads was used to perform read trimming with Canu (v2.2) (Koren *et al*. 2017) and the final hybrid assembly was performed using Flye (v2.9.2) (Kolmogorov *et al*. 2019). Raw nanopore reads were aligned to the assembly for manual inspection using Minimap2 (parameters: -ax -map-ont) (Li, 2018). Subsequent polishing was carried out with Illumina reads using Pilon software (v1.24) (Walker *et al*. 2014) and the resulting assembly was used as a reference for another round of polishing with Pilon (Walker *et al*. 2014). Further rounds did not improve the assembly.

Chromosome-level assembly was conducted using Reference-Assisted Genome Ordering Utility (Ragout) (Kolmogorov *et al*. 2014, 2018). Ragout leverages phylogenetic information to reconstruct probable chromosome rearrangements for the target genome, a methodology previously employed in diverse studies (Andras *et al*. 2020; Corbo *et al*. 2022; Theelen *et al*. 2022). The chromosomes of ARSEF 4556 as well as the final V275 assembly produced by Flye were used as the reference genome and target assembly, respectively. The genome of V275 was submitted to the NCBI Genome Databank under BioProject Number PRJNA1057712 and Assembly Accession number GCA_039795395.1.

### Genome annotation

All assembly annotations were performed using GenSAS v6.0 pipeline, unless otherwise stated. Low complexity regions and repeats were detected and masked using RepeatModeler (v2.0.1) (Flynn *et al*. 2020) and RepeatMasker (v4.1.1) (Smit *et al*. 2021), setting the DNA source to fungi and the speed/sensitivity parameter to slow. A masked consensus sequence was generated, on which *ab initio* gene prediction was performed using GeneMarkES (v4.48) (default parameters) (Ter-Hovhannisyan *et al*. 2008), Augustus (v3.4.0) (Species: *Fusarium_graminearum*, Report genes on_strand: Both, allowed gene structure: Allow prediction of incomplete genes on the sequence boundaries, and using the soft masked sequence) (Stanke *et al*. 2006) and GlimmerM (v2.5.1) (Kelley *et al*. 2012). BLASTn and DIAMOND were used for DNA and protein alignments, respectively. By combining *ab initio* gene predictions, as well as protein and nucleotide alignments, EvidenceModeler was employed to create the consensus gene set. After running BUSCO analyses, the official gene set was produced using GeneMarkES (Ter-Hovhannisyan *et al*. 2008). Ribosomal RNAs (rRNAs) were detected using RNAmmer (v1.2) (Lagesen *et al*. 2007) and tRNAs were determined using tRNAscan-SE (v2.0.7) (Chan and Lowe 2019). In addition, the completeness of genome assembly and protein set was assessed using BUSCO (v5.4.7) (Manni *et al*. 2021), with the assembly and predicted protein sequences of V275 and ARSEF 4556 as respective inputs. BUSCO analyses were performed by comparing against conserved orthologues from the Hypocreales_odb10, Pezizomycotina_odb10, and Ascomycota_odb10 lineages. Results were simplified into categories of complete and single-copy, complete and duplicated, fragmented, or missing BUSCOs.

The mitogenome was annotated using GeSeq (v2.03) (Tillich *et al*. 2017). Basic Local Alignment Tool (BLAT) reference sequences were GenBank files containing mitogenome annotations for *M. anisopliae* (NC_008068.1) and *Metarhizium rileyi* (NC_047289.1). The mitogenome was visualized using OGDRAW (Greiner *et al*. 2019).

### Comparisons of genome structure and synteny

OrthoFinder (v2.5.5) (Emms and Kelly 2019) was employed to determine the orthologous genes of the 2 genomes and separate them into groups. i-ADHoRe 3.0 (Proost *et al*. 2012) was used for the alignment of the orthologous genes and based on their synteny, homologous segments that showed conserved gene order and content were created. Circos (Krzywinski *et al*. 2009) was used to visualize the conserved regions of the genomes. The same approach was also employed with the pseudo-chromosome assembly created in Ragout, to visualize chromosome-based comparisons.

### Functional annotation

Predicted protein sequences were aligned to the functional databases Swiss-Prot using blast2GO suite (Conesa *et al*. 2005), Interpro (v5.53-87.0) and pfam (v1.6) as well as cluster of orthologous Genes (COG) database. using COGclassifier (Shimoyama 2022). Functional annotation was additionally performed using KEGG database, and tools Blastkoala and Ghostkoala, to perform GO and KEGG metabolic pathway enrichment analyses. Proteins encoding signal peptides (secretory or transmembrane) were identified using SignalIP (as implemented in GenSAS). The potential pathogenic and virulence-associated genes were identified by sequence alignment (BLASTp) against the pathogen–host interaction database (v3.5) (PHI-base) (Winnenburg *et al*. 2006), while transporter proteins were predicted using the Transporter Taxonomy Database (Saier *et al*. 2006). Proteases were identified and classified into families by BLASTp (Altschul *et al*. 1997) against the MEROPS peptidase (http://www.ebi.ac.uk/merops) database (Rawlings *et al*. 2016). To explore the genetic potential of these fungi for secondary metabolite production, the cluster predictor AntiSMASH fungal (v.7.0.1) (Blin *et al*. 2023) was employed. Both genomes were screened for the presence of genes and clusters responsible for the biosynthesis of secondary metabolites. Genome sequence fasta files as well as gff gene prediction files from EvidenceModeler were used as inputs, using the default (relaxed) search parameter. All additional features were set to on, including cluster-border prediction based on transcription factor binding sites (CASSIS). An additional similarity network analysis was employed to investigate the similarity of BGCs between the 2 strains, as well as with other plant-associated fungi, using the program Big-SCAPE (v1.1.8) (Navarro-Muñoz *et al.* 2020). All reference BGCs found in MIBiG database were included to identify similarities with known products (Terlouw *et al*. 2023). Search for CAZy enzymes was performed using dbCAN3 server (Zheng *et al*. 2023), which performs automatic CAZyme annotation using Diamond-CAZy, HMMER-dbCAN-substrate, and HMMER-dbCAN-tools. Results supported by at least 2 tools were considered valid and were used for further analyses. The conserved domains of the predicted CAZymes were also characterized using CDD database by NCBI.

To identify orphan genes and determine possible horizontal gene transfer (HGT) events, the database NR was employed and tBLASTn was performed with all V275 proteins as queries against the full nucleotide sequences for all taxa in the *Metarhizium* genus (taxid: 5529). Currently, there are 25 genomes publicly available. This BLAST method was preferred over BLASTp, to take into consideration coding regions that may not yet have been annotated. The results were filtered using an in-house script that evaluates these alignments based on identity and coverage criteria, collecting significant alignments that satisfy a criterion of percentage identity multiplied by coverage surpassing 45%. Consequently, proteins were categorized based on their occurrence either in *M. brunneum* V275 exclusively, additionally in *M. brunneum* ARSEF 4556, *M. brunneum* ARSEF 3297, or in other *Metarhizium* spp. (among the 25 available).

### Phylogeny

Phylogenetic analysis was performed using PhyloBUSCO. The tree was constructed using BUSCO-based analysis on the proteomes of 15 *Metarhizium* strains available in Uniprot database. Predictions were performed on each proteome using BUSCO (v.5.0) (Manni *et al*. 2021) and OrthoDB (v.10) (Zdobnov *et al*. 2020). Sequence alignments were performed using Muscle (Edgar 2004) and trimAl (Capella-Gutierrez *et al.* 2009). Maximum likelihood (ML) tree was inferred using IQ-TREE (v.1.6.12) (Nguyen et al. 2014; Trifinopoulos *et al*. 2016) with the model selection from ModelFinder (Kalyaanamoorthy *et al*. 2017) using the following defaults parameters: “-bb 1000 -alrt 1000 -nt AUTO -ntmax”. The tree file was visualized using the environment for tree exploration Toolkit (Huerta-Cepas *et al*. 2016).

## Results

### General genome features of de novo assembly

Prior to assembly of the V275 genome sequence, relative genome size estimation using k-mer analysis of the raw Illumina sequencing data predicted a genome size of 39.8 Mb with 0.019% heterozygosity (a characteristic “single peak” in the k-mer frequency distribution) and 4.2% overall repeat content. Nanopore sequencing resulted in 1830 fast5 files and “271089244728” samples were base called to an output of 20.3 Gb fastq files (500 × initial theoretical coverage). After error correction, read trimming and polishing, the final hybrid assembly pipeline resulted in 31 contigs (including the contig for the mitochondrial genome) of a cumulative length of 40,108,809 bp (final assembly's mean coverage of 48×) and N50 contig length of 4,322,865 bp, with the largest fragment being 8,292,426 bp was found (Table 1). Moreover, assembly integrity, measured by calculating the number and percentage of complete BUSCOs, showed a high level of completeness of the final assembly, as well as of the predicted protein set (Table 1) with 4,364 of 4,494, 1,665 of 1,706 and 746 of 758 complete gene copies conserved among hypocreales_odb10, ascomycota_odb10, and fungi_odb10 lineages, respectively. Assembly metrics and general genome features of *M. brunneum* V275 and ARSEF 4556 are presented in Table 1 and Supplementary Table 1.

**Table 1.** General genome features of *M. brunneum* V275 (V275) and *M. brunneum* ARSEF 4556 (4,556).

Genome features	V275	ARSEF 4556
Genome size	40,058,873	37,746,951
% GC (Guanine-Cytosine) content	50.75%	50.40%
Contigs	30	7
Mean coverage	48×	100×
Contig N50	4,322,865	4,800,000
Genes	11,776	11,406
Proteins	11,769	11,405
tRNA/rRNA genes	141/30	149/29
Mt genome size	24,966	24,965
Intergenic regions (bp)	19,228,384 (48%)	18,062,245 (47.9%)
Exons	17,006,338 (42.4%)	16,313,808 (43.2%)
Introns	1,871,758 (4.7%)	1,764,317 (4.7%)
Transposable elements	1,977,363 (4.9%)	1,631,546 (4.3%)
BUSCO (fungi_odb10)	98.4%	98.2%
BUSCO (ascomycota_odb10)	97.6%	97.2%
BUSCO (hypocreales_odb10)	97.2%	96.6%
BUSCO protein (hypocreales_odb10)	99.1%	99.1%

**Fig. 1.**
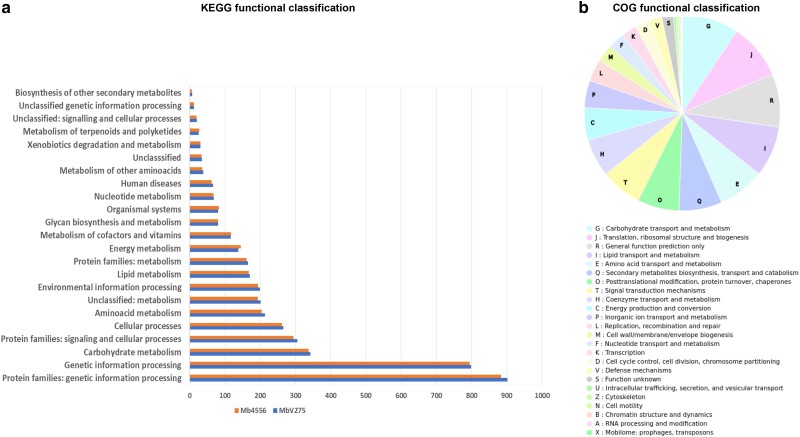
Functional classification of predicted proteins. a) A total number of 4,004 and 3,935 proteins of V275 and ARSEF 4556, respectively, were assigned in 23 functional categories in KEGG database. b) COG annotation classified 4,681 proteins of V275 into 26 COG functional categories.

Genome annotation predicted a total of 11,763 proteins, a slightly higher number than those of ARSEF 4556 (Saud *et al*. 2021). 1,336 proteins had a secretory signal domain (Supplementary Table 2), and 2,981 transporters (Supplementary Table 3) were identified using a 1 × 10^−5^ threshold. The number and types of proteases of both genomes were identified (Supplementary Table 4). Protein assignment of accession numbers from Interpro and Pfam databases was obtained (Supplementary Table 4). KEGG orthology assignments classified 4,004 (30.2%) of V275 proteins into 23 functional categories, with the highest abundance of genes assigned to genetic information processing, carbohydrate metabolism, signaling and cellular processing, and amino acid metabolism (Fig. 1; Supplementary Table 5). ARSEF 4556 proteins were classified into the same categories with minimal differences in the number of proteins in certain categories (Fig. 1; Supplementary Table 5). KEGG Mapper Reconstruction Results showed that proteins were involved in 410 metabolic pathways and 83 modules (Supplementary Table 5). COG annotation showed that 39.79% (4,681 of 11,763) of the total proteins were classified into 26 COG functional categories (Fig. 1; Supplementary Table 5). The tRNAscan-SE tool predicted a total of 124 tRNA genes and RNAmmer predicted a total of 30 rRNA genes present in the genome assembly. The assembly process produced a complete circular mitochondrial genome of 24,966 bp, containing sequences that encode 25 tRNAs, 2 ribosomal RNA subunits, and the 14-core protein-coding genes (Supplementary Fig. 1). As expected with previous findings regarding variations in mt genomes of *Metarhizium* spp. (Kortsinoglou *et al*. 2020), mitochondrial genomes of the 2 strains of the same species present minimal differences.

Interestingly, V275 exhibited some structural peculiarities. Contig 25 (of the 30 assembled chromosomal contigs) showed sequence identity with the plasmid pECQ4552_IHU08 (NCBI Accession number: CP077071.1), previously described in *Escherichia coli* strain Q4552 (Hamame *et al*. 2022). This plasmid was not identified as circular, aligning with similar findings in a *Klebsiella* sp. strain by Sushenko *et al*. (2022). Moreover, it encompassed 7 genes encoding a YlcI/YnfO family protein, a hypothetical protein, a DUF1398 domain protein, a serum resistance lipoprotein Bor, a glycoside hydrolase family protein with a conserved domain of muramidase (phage lambda lysozyme; cl44109), and a prophage endopeptidase RzpD (NP_415088.1). The endopeptidase, originating from a bacteriophage, featured a phage lysis conserved domain (pfam03245) implicated in host lysis and a signal peptide, indicative of its potential involvement in membrane targeting or extracellular secretion. To date, a similar plasmid has not been found in *M. brunneum* ARSEF 4556, as well as in all the other whole genome sequences available from strains of genus *Metarhizium*.

Furthermore, 3 additional fragments of 1.9 (contig 19), 19.19 (contig 21), and 20.92 (contig 2) kb, exhibited significant similarity with the genome of the mutualistic endophyte *Epichloë glyceriae* E2772 (coverage/identity was 71/87%, 88/88.21%, 43/91.68%, respectively), as confirmed by comparison with the NCBI nr/nt database using a stringent percentage similarity filter. Intriguingly, these fragments were not fully identified in other *M. brunneum* genomes (including ARSEF 4556), except for species of *Metarhizium acridum*, albeit, with lower percentages of similarity (20/86, 23/88, and 20/88% coverage/identity, respectively). The first of these fragments contained a single gene matching with *E. glyceriae* strain E2772 (coverage 94%/identity 87%), *M. album* ARSEF 1941 (coverage 100%/identity 76%), and *M. acridum* CQMa 102 (coverage 100%/identity 81%) strains. The second fragment comprised 5 predicted genes that encoded hypothetical proteins without a conserved domain and therefore have yet unknown functions. The third fragment contained 4 genes, out of which only one contained a conserved domain. It corresponded to an orsellinic acid biosynthesis cluster protein (OrsD) found in *Emericella nidulans*. This gene was also found in *M. anisopliae* (Acc. No: AF291909.2), where it is termed as *M. anisopliae rec*Q helicase gene (coverage 100%/identity 95%), and the rhizosphere-associated species *Ilyonectria robusta* (coverage 93%/identity 71%).

### Phylogeny and mating type genes

**Fig. 2.**
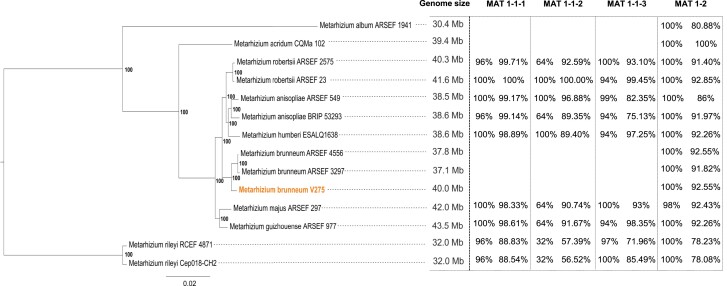
Phylogenetic tree of *Metarhizium* strains. All the available proteomes of *Metarhizium* strains in Uniprot database were used. ML tree was constructed using the BUSCO dataset using Hypocreales_odb10 lineage. Numbers correspond to bootstrap values. Protein similarity search (Blastp) was performed for the detection of MAT genes, using *M. robertsii* strain ARSEF23 MAT1-1-1 (EFZ01122), *M. anisopliae* isolate Ma69 MAT1-1-2 (BAE93597), MAT1-1-3 (BAE93596), and *M. acridum* strain CQMa 102 MAT1-2 (EFY86728) proteins as queries.

The phylogenetic tree showed that V275 strain is basal to the other 2 *M. brunneum* strains ARSEF 3297 and ARSEF 4556 with excellent bootstrap support (100%), but altogether comprise the *M. brunneum* species with 100% bootstrap support (Fig. 2). Strain V275 includes a complete MAT1-2 gene at Contig 5. ARSEF 4556 and ARSEF3297 present a similar MAT gene content, indicating thus, that *M. brunneum* has solely the MAT1-2 gene. Strains of *M. rileyi*, *M. majus*, *M. anisopliae*, and *M. humberi* were all found to harbor both MAT1-1 and MAT1-2 genes. A sole MAT1-2 gene was also found in *M. acridum* and *M. album* strains. Interestingly, *M. brunneum* is phylogenetically placed as a sister group to the subclade of *Metarhizium robertsii*, *M. anisopliae*, and *M. humberi* that present both MAT1-1 and MAT1-2 genes, suggesting a putative homothallism, despite having no known teleomorph (Pattemore *et al*. 2014).

### Chromosome scaffolding as defined by “pseudo-chromosome” analysis—synteny

**Fig. 3.**
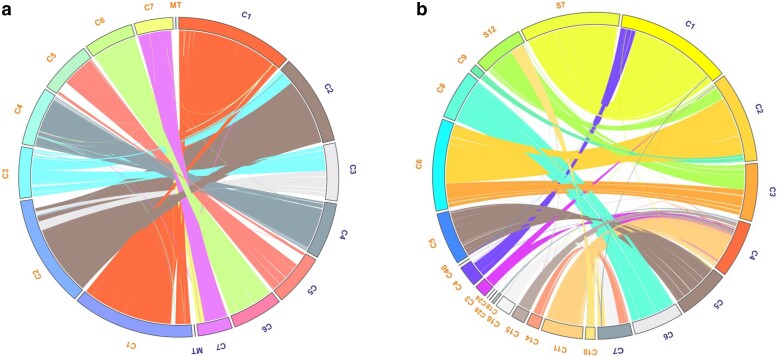
Genome synteny comparison between V275 and ARSEF 4556. a) Comparison using ARSEF 4556 chromosomes and mt genome (blue) and V275 pseudo-chromosomes and mt genome (orange). b) Comparison between ARSEF 4556 chromosomes (blue) and V275 contigs and scaffolds (orange).

Seven pseudo-chromosomes, assembled out of the 30 V275 contigs, were identified using Ragout software (Fig. 3). Due to the current availability of only one *M. brunneum* strain (ARSEF 4556) at chromosome-level assembly and given that pseudo scaffolding led to a lower BUSCO assessment score (97%), the reference-based assembly created by Ragout software was used only for visualizing genome synteny and rearrangements. It was evident, however, that except for three chromosomes of ARSEF 4556, i.e. C1, C3, and C6, which seem identical, excluding unique additional regions seen in the V275 sequence; the rest of the chromosomes present several different syntenic rearrangements that indicate intraspecies variations (Fig. 3).

### Characterization of selected functionally important gene groups

#### Predicted genes for synthesis of volatile organic compounds

Two functionally important volatile organic compounds (VOCs) are produced by *M. brunneum*, 1-octen-3-ol and 3-octenone (IUPAC synonym: octan-3-one) (Wood *et al*. 2022, 2023). In the V275 genome, there were 4 predicted genes encoding a putative protein needed for the deoxygenation of linoleic acid, the first step on 8-C VOC production (Mb.00g043120.m01, Mb.00g025310.m01, Mb.00g090640.m01, and Mb.00g004080.m01 and each showed identity > 98.5% with previously reported dioxygenase genes of *M. brunneum* and *M. anisopliae*) and only one fatty acid hydroperoxide lyase (Mb.00g106540.m01) which was orthologous (99 – 100%) with respective proteins from genomes of *Metarhizium* species. Five putative genes showed similarity to the conserved domain of enone reductase for the final production of 3-octanone, but a full gene orthologue could not be confidently identified.

#### Carbohydrate-active enzymes

**Fig. 4.**
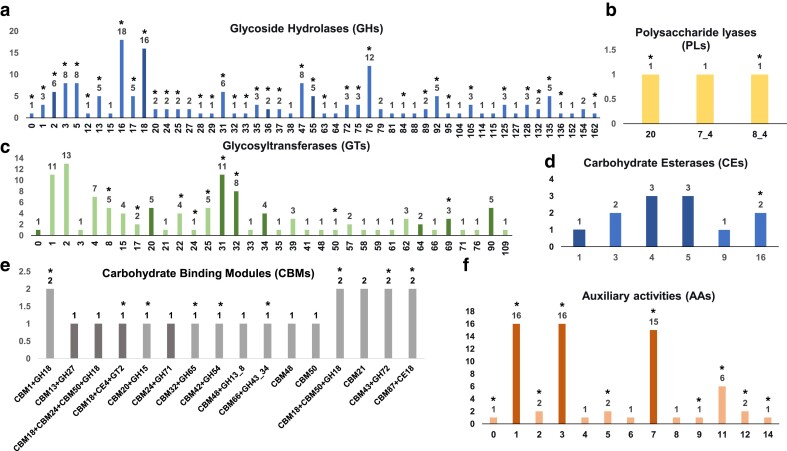
a–f): The CAZy families (*x* axis) and the number of V275 enzymes (*y* axis) belonging to each CAZ enzyme type. Asterisk shows predicted signaling domains in enzymes of this family. Bolder shades indicate different numbers of enzymes between the two strains.

The genome of *M. brunneum* V275 contained 391 genes encoding CAZ enzymes (predicted by 2 or more tools on dbCAN3), responsible for either the assembly (glycosyltransferases, GTs), or the breakdown (CEs, carbohydrate Esterases; PLs, polysaccharitic lyases; and GHs, glucosyl hydrolases;) of carbohydrate complexes (Fig. 4). In addition, enzymes with auxiliary activity (AAs) and 11 different carbohydrate binding modules (CBMs) were described (Fig. 4). Differences with ARSEF 4556 were minimal, with the latter strain containing 389 CAZymes belonging to the same main families (Supplementary Table 6).

In detail, CAZyme annotation revealed that the genome of V275 contains 113 GTs dispersed in 34 families, which are involved in the biosynthesis of oligosaccharides, polysaccharides, and glycoconjugates (Breton *et al*. 2006). Families GT2, GT1, and GT31 are overrepresented with more than 10 members each, while half of GT families (17 out of 34) have only 1 or 2 members.

**Fig. 5.**
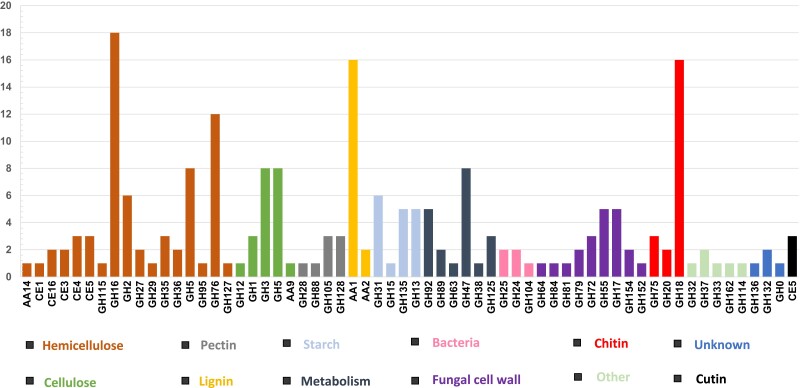
The number (*y* axis) of CAZ enzyme families of V275 strain (*x* axis) and their respective substrates.

Additionally, CAZyme analyses showed that V275 genome contains 169 GHs that belong to 59 families (Fig. 4). Several chitin-targeting GHs were found, as expected considering the entomopathogenicity of these fungi. All other encoded GHs are linked to a range of substrate specificities, including cellulose and cellobiose, hemicelluloses, pectins, β-1,3 glucan, starch, cutin, and bacterial proteins (Fig. 5). Out of all the individual families, GH16 (active on β-1,4 or β-1,3 glycosidic bonds in various glucans and galactans), GH18 [catalytically active chitinases (EC 3.2.1.14) and endo-β-N-acetylglucosaminidases (EC 3.2.1.96)], as well as family GH76 (endo α-1,6-mannanase) were the most abundant in this genome (Figs. 4 and 5). V275 genome also encodes an GH114 family enzyme with *endo*-α-1,4-polygalactosaminidase activity. The homolog protein Ega3 from *A. fumigatus* is found to disrupt the formation of microbial biofilms, which is related to its pathogenicity (Bamford *et al*. 2019). Both V275 and ARSEF 4556 genomes encode an endo-β-1,2-glucanase (GH164, EC 3.2.1.71), an enzyme that was primarily found in eukaryotes in the soil fungus *Talaromyces funiculosus* (Tanaka *et al*. 2019). In that fungus, the enzyme was found to hydrolyze linear and cyclic β-1,2-glucans to sophorose. Sophorose is known to be the most potent inducer of cellulases (Sternberg and Mandels 1979; Bazafkan *et al*. 2014), and thus, V275 can putatively hydrolyze plant cellulose as a carbon source. Blastp results of the protein sequence showed a very high similarity (100% identity and > 90% similarity with all the available *Metarhizium* spp. genomes), with all hits being hypothetical proteins. Matches were also found with other endophytic and plant-related fungal hypothetical proteins, such as *Epichloë festucae* FI1 (QPG94255.1), *Moelleriella libera* RCEF4871 (KZZ99403.1), *Purpureocillium lilacinum* (GJN82308.1), *Claviceps africana* (KAG5920958.1), but with lower similarity percentages (71 – 84%). The V275 strain also encodes sialidases that are used to break down sialic acids. V275 carries also a gene encoding N-acetylglucosaminidase, which hydrolyzes N-acetylglucosamine (GlcNAc), an enzyme found as a component within the cell wall of bacteria, chitin in fungi and in the exoskeletons of arthropods (Sullivan *et al*. 1984). The GH92 family has one representative protein in the V275 genome belonging to exo-acting α-mannosidases, with specificity toward α-1,2-, α-1,3-, and α-1,6-linked mannooligosaccharides.

Furthermore, the V275 genome contains 67 AAs, belonging to 13 families (Fig. 4) that mainly consist of ligninolytic enzymes. Most of these enzymes (50 out of 67) belong to families AA1 (EC 1.10.3.2, 1.10.3—multicopper oxidases and laccases), AA3, including cellobiose dehydrogenases (EC 1.1.99.18) that are exclusively found in wood-degrading and phytopathogenic fungi, AA7 (EC 1.1.3.—glucooligosaccharide oxidases), chitooligosaccharide oxidases (EC 1.1.3.-), and cellooligosaccharide dehydrogenases (EC 1.1.99.-) (Figs. 4 and 5).

CEs are enzymes that remove esters from saccharides (Cantarel *et al*. 2009). 12 enzymes classified in 6 families were predicted, including 6 enzymes for cutin (cutinase, EC 3.1.1.74) and chitin (chitin deacetylases, EC 3.5.1.41) degradation, 2 of which would have specificity to acetic ester (acetyl esterases, EC 3.1.1.6), alongside one N-acetylglucosamine-6-phosphate deacetylase (3.5.1.25) which catalyzes the first step in the biosynthetic pathway to amino-sugar-nucleotides and 3 acetyl-xylan esterases (EC 3.1.1.72), with specificity for xylan (Figs. 4 and 5).

The 3 PLs found were equally dispersed in 3 PL families of V275. Families PL8_4 and PL20 are associated with activities of a pectin methylesterase (EC 3.1.1.11), for degradation of plant cell wall component pectin and of an endo-β-1,4-glucuronan lyase (EC 4.2.2.14) which catalyses the depolymerization of linear β-(1,4)-polyglucuronic acid (glucuronan), respectively (Fig. 4). Due to the poor characterization of these enzyme families and the absence of an Enzyme Commission number (EC number), the protein belonging to PL7_4 family was searched in CDD database to assign a function. CDD and NCBI search recognized a conserved alginate lyase that degrades the linear polysaccharide alginate.

### Transposable elements and genomic intraspecies differentiation

**Fig. 6.**
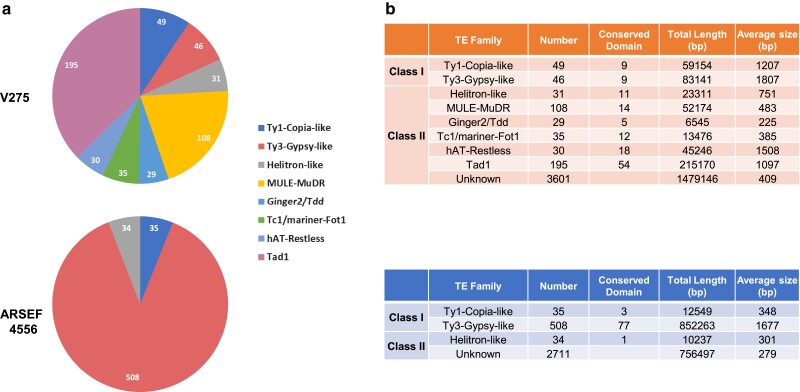
Predicted TEs. a) The number and types of predicted TEs in both genomes of V275 and ARSEF 4556, b) the total number and the number of TEs with conserved domains, their total length and the average size of each predicted TE family in both genomes.

TEs are classified into 2 broad categories: Class I retrotransposons (LTR, SINES, and LINES) and Class II DNA transposons (DNA and Helitron TEs) (Finnegan 1989). RepeatModeler predicted 4,124 (covering 4.9% of the genome length) and 3,129 (4.3%) TEs for V275 and ARSEF 4556, respectively, with most of them in both genomes being of unknown type (Fig. 6; Supplementary File 7). V275 genome was abundant in 8 types of TEs belonging to both Class I RNA transposons (LTR/Copia, LTR/Gypsy) and Class II DNA transposons (Line/Tad1, DNA/MULE, DNA/Ginger-2, DNA/TcMarFot1, DNA/hAT Restless, and RC/Helitron). On the contrary, ARSEF 4556 appeared to have a high abundance of only Class I TEs, an LTR/Gypsy type, and Class IIof RC/Helitron (Fig. 6).

TEs can be further classified according to whether they can move autonomously or not, by encoding the necessary enzymes for their transposition (Wells and Feschotte 2020). Thus, all predicted TEs (excluding the unknown types) were searched in CDD database to locate reverse transcriptase or transposase conserved domains indicative of putatively active mobile TEs. Conserved domains were identified in a relatively small proportion of each TE type (Fig. 6—overall 28% for V275 and 9% for ARESF 4556). The remaining TEs were either too small or did not contain a conserved domain, representing remnants of a previous transposition (Supplementary Table 7).

**Fig. 7.**
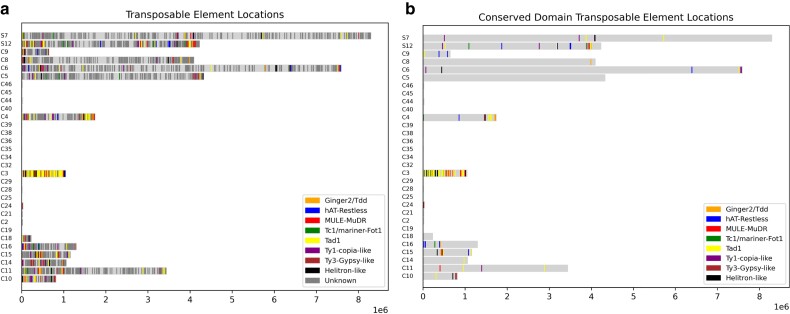
Predicted TEs. a) Location of predicted TEs in each contig of V275, b) location of predicted TEs with a conserved domain in each contig of V275.

An additional examination of the insertion preference of these mobile TEs was performed to assess whether insertion tended towards regions that would not disrupt genes associated with cell function (Fig. 7; Supplementary Table 8). The presence of unknown TEs was widespread across contigs of V275 and TEs with conserved domains did not show a clustering pattern. Contig 3 of V275 genome displayed a notable density of TEs, averaging approximately one transposon per 16 kb, and contained genes with TE-mediated transfer potential (Fig. 7). There were several cases of TEs belonging to all types which were located next to unique genes of *M. brunneum* V275, within the *Metarhizium* lineage (Supplementary Table 8—red color), with others only detected within *M. brunneum* strains (Supplementary Table 8—green).

V275 harbored 108 MULE/MuDR TEs with 14 having a conserved transposase domain, compared with none in ARESF 4556. One MULE/MuDR TE (no 18 of TEs presented in the Supplementary Table 8) was identified between a purple acid phosphatase involved in Phosphorus (P) mobilization from organic compounds and an major facilitator superfamily (MFS) transporter, as well as a *M. brunneum* lineage-specific serine/threonine kinase and a ferric reductase gene. A second Mule/MuDR TE (no 19; Supplementary Table 8), not found in other *Metarhizium* strains, was identified next to a patatin-like phospholipase plant gene, which was also unique to V275. This TE presents a high identity with a respective Mule sequence from *P. chlamydosporia*, *P. lilacinum*, and *Epichloë* sp., other entomopathogenic and plant-related fungi, but not any other *Hypocrealean* species. Another TE (no 20; Supplementary Table 8) was found next to a gene encoding 3 domains, an integrase (pfam00665), which mediates integration of a DNA copy of the viral genome into the host chromosome, an ASF1-like histone chaperone (cl22451) and a gag-polypeptide of LTR copia type (cl26047).

Overall, 6, 2, and 3 genes unique to V275 were located next to Line/Tad, LTR/Gypsy, and Rc Helitron TEs, respectively (Supplementary Table 8).

#### Pathogenicity-related genes

Comparative analysis with the PHI database was conducted to elucidate common and different potential virulence factors of the 2 strains. In V275, a total of 5,250 proteins (*e*-value cutoff 1 × 10^−5^) exhibited similarity with experimentally verified pathogenicity-associated genes in other fungi. These proteins were linked to diverse activities, including reduced virulence (48%), unaffected pathogenicity (37%), loss of pathogenicity (6%), lethality (4%), increased virulence (3%), and some classified as effector-plant avirulence determinants (2%) (Supplementary Fig. 2). The identified genes originated from various modes of life, spanning entomopathogens (e.g. *M. robertsii* and *B. bassiana*), phytopathogens (e.g. *Fusarium oxysporum*, *Verticillium dahliae*, and *Ustilago maydis*), and human pathogenic fungi (e.g. *Cryptococcus gattii* and *Candida glabrata*). Additionally, similarities were observed with pathogenesis-related genes of bacteria and parasites (e.g. *E. coli* and *Trypanosoma* sp.). Detailed information for each PHI gene is provided in Supplementary Table 9.

The comparison with predicted genes of ARSEF 4556 revealed many common proteins, yet 82 and 79 unique pathogenesis-related genes were identified in the genomes of the V275 and ARSEF 4556 strains, respectively. Among these, only 37 out of 82 genes in V275 had conserved domains (Supplementary Table 9), while the remaining were hypothetical proteins. Notably, several of these genes exhibited similarity with genes from phytopathogenic microbes, such as a nitrate/nitrite transporter (*ntr1*) implicated in increased virulence of tomato (*Solanum lycopersicum*) by *F. oxysporum* (similarity 87%) (Gomez-Gil *et al*. 2018), a reductase protein (MoARG5,6) implicated in loss of pathogenicity of *Magnaporthe oryzae* in barley (*Hordeum vulgare*) (Zhang *et al*. 2015), a serine/threonine protein kinase (*cocbk1*) linked to virulence in *Colletotrichum orbiculare* (similarity 74%) in cucumber (*Cucumis sativus*) (Kodama *et al*. 2017), an RXLR effector gene (*SFI4*) of *Phytophthora infestans* (similarity 43%) associated with increased virulence in *Nicotiana benthamiana* (Zheng *et al*. 2014), an FKBP-type peptidyl-prolyl *cis*–*trans* isomerase (BcPIC5) (similarity 67%), responsible for protein folding and posttranslational modifications, which was found to cause reduced virulence of *Botrytis cinerea* in tomato (Gioti *et al*. 2006), and an AM toxin synthase (similarity 46%), related to the pathogenicity of apple tree (*Malus domestica*) by *Alternaria alternata* (Johnson *et al*. 2000). The latter protein possesses a conserved domain of nonribosomal peptide synthetase component F (implicated in secondary metabolites biosynthesis, transport, and catabolism).

Some interesting cases from ARSEF 4556 include similarity with a gene responsible for loss of pathogenicity (*fga2*) seen in *F. oxysporum* against tomato plants (similarity 80%) (Jain *et al*. 2005), a transcription factor (*GzHOMEL026*) causing lethality in wheat by *F. graminearum* (68%) (Son *et al*. 2011), as well as the transcriptional regulator *ZtRlm1* (Mohammadi *et al*. 2020) and the ligase *Myco4* (Yemelin *et al*. 2017), implicated in reduced virulence of *Zymoseptoria tritici* in wheat (similarity 60 and 67%). Even though the domains of the PHI genes have been retained, as this in silico study showed, experimental verification is still required to ascertain their expression.

Focusing on genes experimentally verified in mutualistic endophytes, a great similarity is exhibited between genes implicated in fungal colonization of *E. festucae*. More specifically, V275 encodes genes with 71, 69, and 77% similarity with genes *noxA*, *noxB*, and *noxR*, respectively necessary for the development of appressorium-like hyphae termed as expressorium, for the establishment of an epiphyllous net on the plant host and exiting leaf tissue. In addition, genes associated with the lethality of plant hosts were detected in V275 genome, such as the transcription factor *GzOB030* (97%) (Son *et al*. 2011) and *FgVPS2* (94%) which is part of the endosomal sorting complex (Xie *et al*. 2019).

#### Biosynthetic gene clusters

**Fig. 8.**
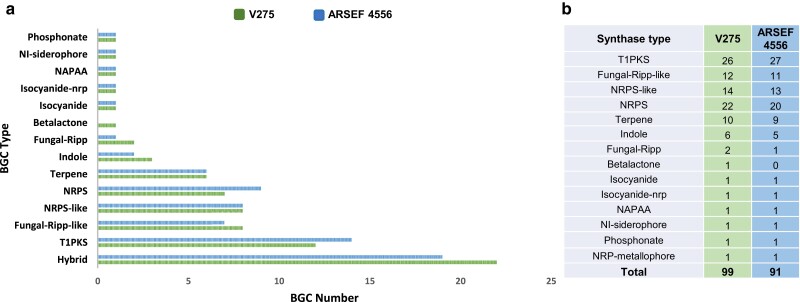
a) The number and type of BGCs located in ARSEF 4556 (in blue) and V275 (in green) genomes, b) the number of synthases of each type.

Cluster prediction using AntiSMASH showed that V275 and ARSEF 4556 exhibit a variety of putative biosynthetis gene clusters (BGCs), encoding 74 and 71 clusters, respectively. Predominantly, these clusters feature type i polyketide synthase backbones (T1PKS), although a diverse array of 10 different classes of nonribosomal peptide synthetases (NRPS), NRPS-like, terpene, indole, Fungal-RiPP-like, and hybrid clusters (harboring combinations of 2 or more backbones) were also detected (Fig. 8; Supplementary Table 10). The genome of ARSEF 4556 has more NPRS, T1PKS, and terpene clusters, whereas V275 has more indole, Fungal-Ripp, Ripp-like and hybrid clusters (Fig. 8). Both genomes contain a high number of genes corresponding to synthases for each BGC type, with V275 presenting a higher number in 6 out of 14 synthase types (Fig. 8b). Intriguingly, only around half of the total predicted V275 and ARSEF 4556 BGC clusters could be linked to a known product (Table 2), while the rest are cryptic.

**Table 2.** Predicted BGCs associated with known compounds.

Compound	MIBiG accession	Biosynthetic class	Cluster type	% Identity—ARSEF 4556	% Identity—V275	Organism	Reference doi
Viridicatumtoxin/previridicatumtoxin/5-hydroxyanthrotainin/8-*O*-desmethylanthrotainin	BGC0000168	Iterative PKS1	T1PKS	40	40	*Penicillium aethiopicum*	10.1016/j.chembiol.2010.03.015
CIML B/CIML A/CIML D/CIML C	BGC0002228	NRP	T1PKS	100	100	*Colletotrichum incanum*	10.1021/acs.orglett.0c01975
Choline	BGC0002276	NRP	NRPS-like	100	100	*Aspergillus nidulans FGSC A4*	10.1073/pnas.1903282116
Enniatin	BGC0000342	NRP	NRPS-like	100	100	*Fusarium equiseti*	10.1111/j.1365-2958.1993.tb01181.x
metachelin C/metachelin A/metachelin A-Ce/metachelin B/dimerumic acid 11-mannoside/dimerumic acid	BGC0002710	NRP	NRPS	100	100	*M. robertsii ARSEF 23*	10.3389/fmicb.2021.783609
Peramine	BGC0002164	NRP	NRPS	100	100	*E. festucae*	10.1111/j.1365-2958.2005.04747.x
Clapurines	BGC0001365	NRP	NPRS, Indole	100	100	*Claviceps purpurea 20.1*	10.1371/journal.pone.0158945
ε-Poly-L-lysine	BGC0002174	NRP	NAPAA	100	100	*E. festucae*	10.3390/molecules25051032
Destruxin A	BGC0000337	NRP	hybrid (NRPS, T1PKS)	61	57	*M. robertsii ARSEF 23*	10.1006/jipa.1999.4884
Ochratoxin A	BGC0002605	NRP + polyketide	NRPS, T1PKS	40	40	*Aspergillus carbonarius*	10.1016/j.ijfoodmicro.2017.12.028
Monoascorubin	BGC0000099	NRPS	NRPS	841	100	*Talaromyces marneffei*	10.1038/srep06728
Serinocyclin A/Serinocyclin B	BGC0001240	NRPS	NRPS	100	100	*M. anisopliae*	10.1021/np070407i
Pyrichalasin H	BGC0001881	NRPS/Iterative PKS1	T1PKS, NRPS	45	36	*Pyricularia grisea*	10.1111/j.1364-3703.2005.00309.x
Swainsonine	BGC0002270	NRPS/Polyketide	NRPS-like, T1PKS	85	85	*M. robertsii ARSEF 23*	10.1021/acschembio.0c00466
BII-rafflesfungin	BGC0001966	NRPS/Polyketide	Fungal-Ripp-like, NRPS	15	15	*Phoma* sp.	10.1186/s12864-019-5762-6
BAB/BAA	BGC0002240	Polyketide	T1PKS,NRPS-like	100	100	*M. anisopliae*	10.1016/j.fgb.2021.103568
UNII-YC2Q1O94PΤ	BGC0001252	Polyketide	T1PKS, NRPS	—	100	*A. alternata*	10.1094/MPMI-06-12-0155-R.
Ustilaginoidin N/ustilaginoidin O/ustilaginoidin M/ustilaginoidin A/ustilaginoidin F/ustilaginoidin E/ustilaginoidin D/ustilaginoidin G	BGC0002301	Polyketide	T1PKS, NRPS	46	46	*Aschersonia paraphysata*	10.1111/1462-2920.14572
Burnettiene A/B preburnettiene A/preburnettiene B	BGC0002139	Polyketide	T1PKS	25	25	*Aspergillus burnettii FRR 5400*	10.1039/d1ob01766g
Citreoviridin	BGC0001400	Polyketide	T1PKS	60	60	*Aspergillus terreus NIH2624*	10.1021/acs.orglett.6b00299
YWA1	BGC0002175	Polyketide	T1PKS	100	100	*Aspergillus oryzae RIB40*	10.1186/s12896-019-0567-x
Harziphilone/t22azaphilone/isoharziphilone-1/isoharziphilone-2/compound 4/compound 1	BGC0002206	Polyketide	NRPS, T1PKS	-	40	*Trichoderma guizhouense*	10.1111/1462-2920.15246
Lucilactaene	BGC0002261	Polyketide	NRPS, T1PKS	69	69	*Fusarium* sp.	10.1080/09168451.2020.1725419
Cryptosporioptide B	BGC0002063	Polyketide	NRPS, NRPS-like, T1PKS, fungal-RiPP-like, Terpene	23	23	*Cryptosporiopsis* sp. 8999	10.1039/c8sc05126g
Pyranoviolin A	BGC0001124	Polyketide/NRP	NRPS, T1PKS	25	25	*Aspergillus violaceofuscus*	10.3389/fmicb.2020.562063
Subglutinol A/Subglutinol B	BGC0002427	Polyketide/Terpene	T1PKS, terpene	50	66	*M. robertsii ARSEF 23*	10.1038/s41467-020-15664-4
Phomopsin A/b/E	BGC0001398	RiPP	Fungal-RiPP	14	14	*Diaporthe toxica* (*Phomopsis* sp.)	10.1073/pnas.1522907113
Clavaric acid	BGC0001248	Terpene	Terpene	100	100	*Hypholoma sublateritium*	10.1016/j.fgb.2008.12.002
Eupenifeldin	BGC0001976	Terpene	T1PKS	36	36	*Phoma* sp.	10.1016/j.fgb.2019.04.004
Nivalenol/deoxynivalenol/3-acetyldeoxynivalenol/15-acetyldeoxynivalenol/neosolaniol/calonectrin/apotrichodiol/isotrichotriol/15-decalonectrin/T-2 toxin/3-acetyl T-2 toxin/trichodiene	BGC0001278 (BGC0001277)	Terpene	Phosphonate	8	8	*Fusarium sporotrichioides* (*F. graminearum*)	10.1128/AEM.68.5.2148-2154.2002
Terpendole E	BGC0001260	Terpene	Indole, Terpene	100	100	*Tolypocladium album*	10.1016/j.chembiol.2012.10.010.
Lysergic acid/elymoclavine	BGC0001267	Terpene	Fungal-Ripp-like	23	23	*Claviceps fusiformis*	10.1128/AEM.01040-07
Ergotamine	BGC0001241	Terpene/Alkaloid	Indole, NRPS	58	58	*Claviceps fusiformis/Claviceps purpurea 20.1*	10.1016/j.phytochem.2005.04.011

**Fig. 9.**
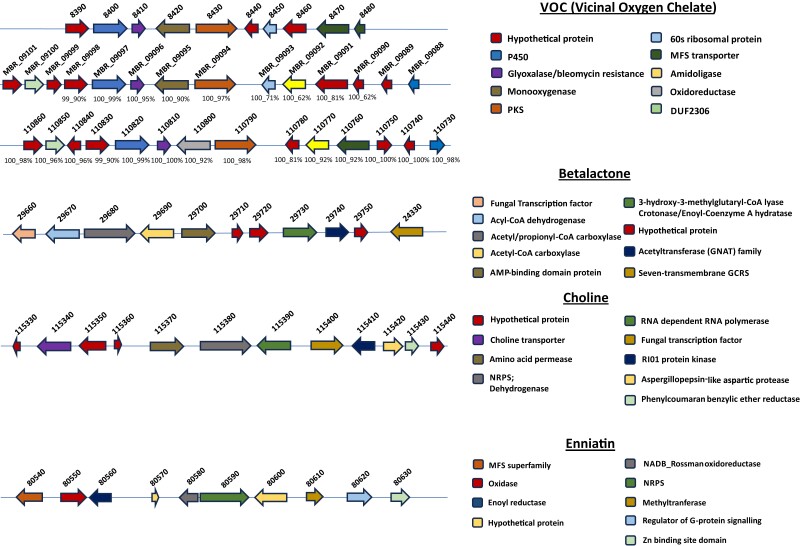
Gene organization of the VOC and betalactone clusters as well as the BGCs related with choline, and enniatins production in V275 strain.

Annotated BGCs of NRPS and NRPS/polyketide type, associated with the production of compounds destruxin A, serinocyclin, and swainsonine, which are insecticidal and mycotoxic compounds of *Metarhizium* spp., were identified in both *M. brunneum* strains and have been previously identified in other EPF strains (Skrobek and Butt 2005; Krasnoff *et al*. 2007; Luo *et al*. 2020). Another important BGC found in V275 genome is that of the NRP cluster of metachelin. It has also been described in *M. robertsii* (Zhang *et al*. 2020), but not in other genomes of the *Metarhizium* genus. Additional NRP clusters encoding antimicrobial CIML compounds (A to C), which are fungal macrolide natural products that exhibit antifungal activity, have also been previously described (Morishita *et al*. 2020), as well as in both V275 and ARSEF 4556 genomes. In *M. brunneum* strains, a newly described BGC comprising 12 genes has been predicted in this study. This cluster includes a choline synthase, a choline transporter, an amino acid permease, as well as a transcription factor potentially regulating choline biosynthesis (Fig. 9). Notably, a protein processing both cytoplasm to vacuole targeting domain and a toxin-10 domain, which is found in insecticidal bacterial proteins, is also part of this cluster (Fig. 9). Besides choline, enniatins was another class of compounds found in V275 and ARSEF 4556 (Fig. 9). In *M. brunneum*, the cluster appears to be comprised of 19 and 20 genes in V275 and ARSEF 4556, respectively. Both *Metarhizium* strains have a cluster associated with the production of a thioclapurine analog from *Claviceps purpurea* (Dopstadt *et al*. 2016). Comparison of synteny and protein sequences of these genes in both clusters showed a high similarity. Remarkably, both V275 and ARSEF 4556 strains harbor 2 BGCs, i.e. peramine and ε-Poly-L-lysine, which are associated with plant colonization. These clusters are involved in insect feeding deterrence and inhibition activity against plant pathogens, during plant colonization of the mutualistic endophyte *E. festucae* (Tanaka *et al*. 2005; Purev *et al*. 2020). The gene involved in peramine biosynthesis, a *per*A synthase orthologue with the same domain order and identity, has also been detected in *M. rileyi* and *M. majus* (Berry *et al*. 2019). Intriguingly, while production of ε-Poly-L-lysine has not been previously reported in the genus, comparison of aminoacid sequences against NR NCBI database revealed the presence of the respective synthase gene (*epls*) in several *Metarhizium* spp., along with several plant-related fungi like *Fusarium* spp., *Emericellopsis* spp. and *P. chlamydosporia* and mycophilic fungi like *Cladosporium mycophilum*. Direct comparison of the protein sequences between *epls* gene of *E. festucae* and the respective gene in V275 exhibited a percent identity of 65% with 98%, coverage, indicating the presence of an orthologue gene.

Polyketide clusters that have been detected in both strains and have been previously reported in *Metarhizium* include the cluster responsible for the production of BAB/BAA compound (Sbaraini *et al*. 2021). V275 also harbors polyketide clusters that are associated with phytotoxic compounds (Table 2). A characteristic example is the cluster responsible for the compound UNII-YC2Q1O94PΤ (synonyms: ACR-toxin I) production. Initially characterized in *A. alternata*, this cluster includes the homologous gene ACRTS2, pivotal for biosynthesis of host-selective ACR-toxin in lemon pathotype of *A. alternata* (Izumi *et al*. 2012). The V275 core biosynthetic protein of this cluster shows 46.82% similarity with ACRTS2 and a 99% alignment coverage, suggesting that it is its homolog, and they share the same protein domains, increasing the possibility of producing the same product.

Several terpene clusters were predicted, that had a relatively low similarity with existing compounds (Table 2). However, BGCs of clavaric acid and terpendole E presented a high similarity with the respective clusters from *Hypholoma sublateritium* and *Tolypocladium album*. Terpendole E is a kinesin Eg5 inhibitor, and thus, a potent anticancer drug (Motoyama *et al*. 2012).

A newly characterized class of fungal natural products corresponding to isocyanide synthase (ICS) BGC was also detected. Products of the cluster mediate pathogenesis, microbial competition, and metal homeostasis through metal-associated chemistry (Nickles *et al*. 2023). The main synthase gene in V275 contained 2 conserved domains, i.e. DIT1_PvcA and TauD.

To characterize the unknown BGCs, above mentioned as cryptic, MiBIG cluster family prediction was employed. Clusters encoding the compound UNII-YC2Q1O94PΤ, subglutinol A/B, cryptosporioptide B, monoascorubin, exist in both strains but were placed in different families due to rearrangements. Out of the unknown clusters, 29 were found to be common in both strains and were placed in the same families (Supplementary Table 10). In addition, 8 and 9 unique clusters were detected in V275 and ARSEF 4556 genomes, respectively (Supplementary Table 10). These are not associated with an existing compound, nor do they present syntenic similarity that would justify the production of similar compounds. This is an indication of the diverse secondary metabolic potential of closely related strains of the same species.

cryptic BGCs found in V275 (and its relatives) were further analyzed due to their potential intriguing function: One of the unknown common clusters between the 2 strains contains a gene with vicinal oxygen chelate superfamily domain, offering glyoxalase/bleomycin resistance (Clusters 37_3, 8_5 in Supplementary Table 10) (Fig. 9). This gene was solely found in *M. brunneum* strains ARSEF 4556, V275, and ARSEF 3297 and it seems to be a species-specific gene. Downstream of this gene, a gene encoding a monooxygenase was also detected only in *M. brunneum* strains. This cluster could potentially be used for resistance to antifungal drugs or resistance to the compound produced by the same strains. The function of glyoxalase/bleomycin resistance protein is to ameliorate the toxicity of methylglyoxal, a by-product of glycolysis (Kargatov *et al*. 2018). A respective ARSEF 4556 cluster is grouped in the same family, but V275 cluster appears to be expanded upon with the addition of multiple genes.

The V275 strain also contains a BGC cluster of 8 genes with a core biosynthetic gene related to the production of a nonNRPS related siderophore. Whereas siderophores have been associated mainly with environmental iron acquisition under iron starvation (Chareyre and Mandin 2018), they may be implicated in a variety of other processes (Rütschlin *et al*. 2018). The core biosynthetic enzyme of this cluster in *M. brunneum* belongs to IucA/IucC family related to aerobactin biosynthesis and possesses a RhbC domain. Notably, tblastn analysis reveals the presence of this cluster solely in *M. brunneum* and *M. robertsii* strains among all *Metarhizium* species (similarity above 55%) and it can be detected in taxa within Eurotiales, Agaricales and Hypocreales (specifically, only in *Metarhizium*, *Cladobotryum*, and *Fusarium* spp.) within the Kingdom of Fungi, with lower similarity.

A distinctive feature of V275 is the presence of a betalactone cluster which is absent from ARSEF 4556 (Fig. 9). This 37Kb cluster found in V275 contains 12 genes, including 2 core biosynthetic enzymes and 2 additional biosynthetic genes. It also encodes a fungal-specific transcription factor, proteins implicated in fatty acid biosynthesis and a G protein-coupled receptor that transmits extracellular signals into the cell. Betalactones natural products have been recognized for their potential antibacterial and antifungal activities (Robinson *et al*. 2018). Interestingly, the betalactone cluster has also been found in entomopathogenic nematode-related bacteria where it serves as proteasome inhibitor (Shi *et al*. 2022). This cluster comprises proteins with the same domains as the V275 cluster (acyl-CoA synthetases and transcription factor).

#### Identification of *Metarhizium* strain- and species-specific genes

The search for unique proteins was extended in all the available *Metarhizium* strains. The set of predicted proteins of V275 was searched to detect those that can only be found in *M. brunneum* strains, among all *Metarhizium* species, as well as proteins that are unique to *M. brunneum* V275, compared to all the available *Metarhizium* strains. Results revealed 224 proteins that can only be detected in all *M. brunneum* strains V275, ARSEF 4556, and ARSEF 3297 within *Metarhizium* lineage. Out of these proteins, 104 had either a known conserved domain or a conserved domain of unknown function (Supplementary Table 11). GO term annotation allocated these proteins into nineteen categories based on biological function, with most of the proteins corresponding to transmembrane transport, proteolysis and regulation of DNA transcription (Supplementary Fig. 3). In addition, 28 GO terms were assigned for molecular function, with the majority belonging to oxidoreductase activity, ion binding/monooxygenase activity and protein binding (Supplementary Fig. 3). Interestingly, all *M. brunneum* strains contain a protein with an endotoxin_N superfamily domain (cl04339), which contains insecticidal toxins produced by the bacterial genus *Bacillus* spp. Once activated, the endotoxin binds to the gut epithelium and causes cell lysis leading to death. Another interesting protein belonged to the GRDP-like superfamily (cl42056) which is found in glycine-rich domain proteins of *Arabidopsis*. Besides *M. brunneum* strains, the protein was identified in the Xylariaceae endophyte *Xylaria bambusicola*, Microascaceae mold *Wardomyces moseri,* with around 75% coverage and 43% identity, and in several *Colletotrichum* sp. strains with a slightly lower identity score (37%). This protein is involved in stress responses in *Arabidopsis* plants, since experimental overexpression led to improved stress tolerance and accelerated plant growth, with indications that the auxin pathway may be involved (Ortega-Amaro *et al*. 2015). An additional protein harboring a GH18 chitinase domain, with the additional transcription factor and chitin recognition protein domains, was shared among the 3 strains.

Interestingly, *M. brunneum* V275 was found to harbor 414 unique proteins among all other available *Metarhizium* strains, out of which around half (219) had a conserved domain of a known function. These proteins were categorized in 61 GO terms for biological processes, with most of them belonging to DNA integration and protein phosphorylation (Supplementary Fig. 3). In addition, 55 GO terms were assigned for molecular functions, mainly belonging to protein and nucleic acid binding functions. These proteins are involved in a variety of functions, among which transportation (MFS transporters, multidrug resistance-associated proteins/ABC transporters, and K^+^ potassium transporters) and RGD2-like proteins with RhoGAP (GTPase-activator protein for Rho-like small GTPases) domain, that activate effectors involved in a wide variety of developmental processes. Also identified were HET proteins, protein kinases, a regulatory locus for aflatoxin biosynthesis (AflR) (Woloshuk *et al*. 1994), a patatin-like phospholipase with lipid acyl hydrolase activity, cytochrome P450 superfamily proteins, CAP domain superfamily proteins, which include PR-1, NADP-Rossman superfamily proteins, a jacalin-like plant-associated sugar-binding superfamily protein, an integrase associated with viral DNA integration in to host chromosomes, and transcription factor domain proteins. Moreover, a protein with a heavy-metal-associated domain which is found in several heavy metal transport or detoxification proteins and has been associated with abiotic stress tolerance in *Saccharomyces cerevisiae* (Sun *et al*. 2014) was detected in V275 genome. A protein with aerolysin domain may be implicated in insecticidal activities since members of this family include enterolobin, a cytolytic, inflammatory, and insecticidal protein from the Brazilian tree *Enterolobium contortisiliquum* (Lima *et al*. 1999).

## Discussion

This study presents a comprehensive genome analysis of the highly effective entomopathogenic and endophytic fungal strain *M. brunneum* V275. The analysis includes a detailed comparative intraspecies genomic investigation, incorporating another interesting for commercial exploitation strain, *M. brunneum* ARSEF 4556, as a reference (Saud *et al*. 2021). Interestingly, V275 possesses the largest genome among *M. brunneum* strains, although it falls within the range of genomes documented for *Metarhizium* species and other Hypocrealean Ascomycetes (Gao *et al*. 2011; Saud *et al*. 2021). Synteny analyses showed a great gene order conservation between the strains, with few rearrangements and some unique fragments. Previous Whole Genome analyses have revealed high levels of synteny between different *Metarhizium* species, such as *M. anisopliae* and *M. acridum* (Gao *et al*. 2011), while the existence of these relocations within *M. brunneum* strains, as well as the unique genome fragments that render V275 genome larger than the ones of ARSEF 4556 and ARSEF 3297, indicate that genomes of this lineage have a dynamic genomic organization, with smaller scale evolutionary events shaping the intraspecies relationships.

### Phylogenetic relationships and MAT gene composition

The tree produced by the ML method (Fig. 2) is in accordance with *Metarhizium* species’ phylogeny proposed by the employment of traditional molecular markers for the taxonomy of the genus, like ITS1-5.8S-ITS2 (ITS) in combination with EF-1, or mitochondrial genes and regions (Bischoff *et al*. 2009; Kortsinoglou *et al*. 2020). Even though the three *M. brunneum* strains group together when compared with the other *Metarhizium* species, strain V275 is placed basally to the other two *M. brunneum* strains. This topology which is based on the BUSCO single-copy protein matrix, indicates that there may be several changes in the amino acid sequences of these proteins which has led to the differentiation of this strain compared to the other 2 *M. brunneum* strains, or vice versa. The higher level of diversification of V275 is also evident by other genomic attributes, since its genome is larger than the respective genomes of the other two strains and thus, it also shows different gene content and synteny (Fig. 3) which might be an indication of carrying some elements, regions and genes which their common ancestor had and were lost during evolution. Another plausible explanation may be the acquisition of the unique V275 genomic fragments through HGT events later in evolution, which leads to an expansion of the genome through interactions with its different hosts and differentiates this genome from the other strains.

The phylogeny of *M. brunneum* may be correlated with the mating type organization of each strain (Fig. 2). *M. brunneum* V275 presents a complete MAT1-2 gene at Contig 5, in a similar gene organization seen in the *M. brunneum* strains ARSEF 4556 and ARSEF3297 genomes (Saud *et al*. 2021). Strains of *M. anisopliae*, *M. guizhouense*, *M. humberi*, *M. majus*, *M. rileyi*, and *M. robertsii* were all found to harbor both MAT1-1-1 and MAT 1-2 genes, in contrast with previous work that supported the concept that *M. majus* would be the sole species that harbors both genes and thus, it is homothallic (Hu *et al*. 2014). Therefore, V275 genome analysis verifies the previous finding that *M. brunneum* along with *M. pingshaense*, *M. acridum* and *M. album* are haploid and presumed heterothallic (Kepler *et al*. 2015).

### Genes implicated in the dual (entomopathogenic and endophytic) mode of life of *M. brunneum* strains

The in silico analyses of V275 and ARSEF 4556 revealed notably elevated BUSCO scores, along with the lowest count of contigs and chromosomes among all currently sequenced *Metarhizium* genomes, respectively. The comparative genome analyses unveiled significant diversity, not only in genome size (∼2 Mb) but also in gene content. While the variation in gene numbers was marginal, the presence of distinct singletons in each genome (Supplementary Table 11) was particularly intriguing. This phenomenon suggests that these singletons might be associated with functions that differentiate the adaptive mechanisms employed by these strains despite sharing similar lifestyles and capabilities (Wood *et al*. 2022). Moreover, the identified genes common to all *M. brunneum* genomes were found to be unique when compared with other taxa of the genus *Metarhizium*, further emphasizing the species-specific genetic repertoire. Therefore, in this study, the finding of several genes and genetic elements may help in the better understanding of the mechanisms employed in entomopathogenicity and endophytism. The origin of these strain- or species-specific genes can only be speculated. Their variation may be associated with TE-mediated transposition, since many of these unique genes were located next to active TEs with conserved domains. They may also be a result of HGT events, or they may even constitute de novo evolved genes. Another possible explanation could be that these genes are fast evolving and thus, show low homology with the respective genes from other strains. To exclude this possibility, we chose to set low similarity criteria during our tBLASTn search. Even though there are strong indications of HGT events in certain cases, an extensive study of their phylogenetic distributions is required to establish the origin of these genes.

Additionally, this analysis presents the first evidence that a plasmid of phage origin exists within the *M. brunneun* V275 genome, probably acquired through a HGT event. Its existence, along with the genes it harbors, indicates its potential involvement in the antimicrobial activity exhibited by the fungus, when found in a soil environment or in a plant or insect host and their indigenous microbiomes. It is well documented that *Metarhizium* species, as well as other entomopathogens and endophytes present an antagonistic behavior against microbes found in the same niches (St Leger and Wang 2020 and references therein). Therefore, this plasmid and its genes may be an additional weapon in the arsenal of the strain for better adaptation to the environment and eliminating antagonism.

Additionally, our detailed analysis of CAZymes revealed a substantial number of CAZymes in both genomes of *M. brunneum* strains, including enzymes necessary for insect pathogenesis, but also numerous plant cell wall degrading enzymes (PCWDEs). Overall, the metabolic enzyme arsenal of *M. brunneum* V275 (Figs. 4 and 5), and similarly of ARSEF 4556, is in accordance with the general notion that substrate metabolism has a vital role in promoting fitness for growth and reproduction and therefore, likely plays a significant role in evolutionary speciation and selection (Hage and Rosso 2021). The diverse range of genes encoding enzymes for degrading various substrates present in *M. brunneum* genomes, underscores their complex ecological roles, since it has been previously shown that the number and diversity of GHs present in fungal genomes are correlated with their lifestyle (Hage and Rosso 2021; Bradley *et al*. 2022). It is estimated that fungus–plant associations originated around 750 million years ago (Douzery *et al*. 2004), and thus, the established association of *Metarhizium* species with plants may predate their entomopathogenic activity (Hu *et al*. 2014). Within the Clavicipitaceae family, recognized for encompassing both plant pathogens and symbionts (Kepler *et al.* 2012), recent estimates indicate that the divergence of the *Metarhizium* from the plant endophyte *Epichloë* lineage occurred ∼ 307 million Years Ago (St Leger and Wang 2020). However, beneficial plant endophytes, like *Epichloë*, typically encode a relatively lower number of PCWDs targeting plant cell walls (e.g. pectinases, cellulases, and hemicellulases) (Hane *et al*. 2020) compared to plant pathogens (Rafiqi *et al*. 2023). Cellulase, xyloglucanase, and pectinase genes were part of the ancestral fungal toolkit, since they were present in early diverging fungi that were associated with streptophytes (Douzery *et al*. 2004; Hage and Rosso 2021) and as shown in this work, they have been retained in the *M. brunneum* genomes (Figs. 4 and 5). Some of these enzymes are related to the activation of plant immunity in plant pathogenic fungi (de Azevedo Souza *et al*. 2017), since cellulases, xylanases, and cutinases have been linked with plant virulent infection of *Fusarium* sp. and *M. oryzae* on wheat and rice, respectively (Kikot *et al*. 2009; Quoc and Bao Chau 2017; Rafiei *et al*. 2021). Moreover, pectin lyases have been linked with the pathogenicity of phytopathogenic fungi such as *Colletotrichum cossodes* and *V. dahliae* (Ben-Daniel *et al*. 2012; Yang *et al*. 2018), and 2 of the 3 V275 pectin lyases had conserved domains and a signal peptide domain for membrane localization or extracellular secretion (Fig. 4). Consequently, the presence of PCWDEs coupled with the overall larger abundance of CAZymes in *M. brunneum*, when compared to grass endophytes, like *Epichloë* spp, challenges their characterization as mutualistic endophytes with beneficial effects on plants. In that sense, the mechanism employed by EPF to avoid triggering plant defense responses during endophytic colonization remains elusive. It is already known that endophytic EPF induce plant systemic resistance through the expression of metabolites (Jaber and Ownley 2018) and the possibility that these plant-degrading enzymes are involved in the mechanism of plant immunity activation described in several works remains to be further explored. However, in this study, several of these CAZ enzymes were found to contain secreted signal domains (Supplementary Table 2), and such enzymes have been linked to the promotion of host colonization and activation of host responses (Kubicek *et al*. 2014). Secreted GH16 enzymes have been associated with translocation into the host and activation of plant defense responses in tomato and *N. benthamiana* (Bi *et al*. 2021). Alternatively, the abundance of PCWDE CAZymes in EPF may be explained by the strains’ potential inability to express these genes or to the level of silencing during their invasion or colonization of their plant host. This aligns with the hypothesis proposing the evolution of endophytism from the saprophytic state of EPF, as these enzymes were essential for substrate decomposition in the soil (Brundrett 2002). The early divergence of the saprophyte and occasional mushroom pathogen species *Metarhizium marquandii* further supports this notion (Rehner and Kepler 2017). However, the absence of a genome sequence for this phylogenetically basal *Metarhizium* species leaves the adoption of different modes of life for EPF unclear. All the above provide indications that comparison of enzymes that fungi secrete can or should be associated with fungal evolution (Barrett *et al*. 2020), since they may provide useful information about the taxonomy and evolutionary relationships of organisms, especially those with multiple modes of life.

Moreover, our study has identified a specific chitinase gene that encodes a GH18 domain. This chitinase is implicated in manipulating a plant's chitin-triggered immunity, as previously documented (Fiorin *et al*. 2018). This discovery indicates that *M. brunneum*, while not posing a threat to its plant host, has potential to confer protection as an endophyte by activating the host plant's defense mechanism. EPF do not trigger plant defense responses towards their own, which indicates that they are not considered a threat (Hao *et al*. 2017). It is also plausible that these genes are remnants of ancestral genes, suggesting the evolution from a plant pathogenic mode of life to a mutualistic one.

The identification of unique V275 genes (among *M. brunneum* strains), such as those encoding patatin-like phospholipases, a jacalin-like superfamily protein, and a protein with a heavy-metal-associated domain typically found in plant genomes, suggests a potential contribution of the fungus to additional biotic and abiotic stress tolerance in their plant hosts, as shown for these genes when located in the plant genome (Gigon *et al*. 2004). Patatin-like PLA_2_ enzymes have been found to act as effector molecules in several pathogenic bacteria to target host cellular membranes (Anderson *et al*. 2014). Although more experimental data are needed, it may be suggested that these genes may render *M. brunneum* a valuable partner for plants, providing adaptive advantages under diverse environmental conditions.

Our analyses of PHI genes revealed a notable proportion of genes associated with arthropod pathogenicity, underscoring the multifaceted nature of these fungi. While earlier investigations in *Metarhizium* spp. suggested a smaller percentage for these genes (Gao *et al*. 2011), it is essential to note the exponential growth in the number of experimentally studied genes implicated in fungal pathogenicity over the past decade (for review see Wang *et al.* 2012 and references therein). For instance, a significant identity (80%) was found with gene so the deletion of which leads to disruption of mutualistic symbiosis with the plant host (Charlton *et al*. 2012). Surprisingly, in several cases, a large similarity against genes associated with reduced virulence of *F. graminearum* was detected. Some of these cases involve *FgAP2mu*, a gene mediating fungal polarity during plant infection (identity 100%) (Zhang *et al*. 2019) and *elp3*, an elongator complex gene involved in the development and oxidative stress response of the fungus (identity 98%) (Lee *et al*. 2014). Consequently, the expanding dataset within the PHI database aligns with the enhanced understanding of the intricate genetic underpinnings governing the pathogenic potential of these fungi.

VOCs are produced by both plants and fungi. In the case of *M. brunneum* it is well established that two VOCs, i.e. 1-octen-3-ol (common name: Mushroom/Matsutake alcohol) and 3-octanone (IUPAC synonym: octan-3-one), are produced and act as biostimulants to the growth of plants (Wood *et al*. 2022) and/or pest repellents, especially for wireworms in small quantities (Wood *et al*. 2023) and lethal in larger concentrations (Khoja *et al*. 2019). Our whole genome analysis showed that *M. brunneum* strains do not contain a BGC cluster related to their production. It is now known that the precursor for both these VOCs is linoleic acid. Linoleic acid is dioxygenased to form 10(s)-hydroperoxide (10-HPOD) from a dioxygenase containing a cytochrome P450-related domain [DOX-(CYP)] and 10-HPOD is subsequently cleaved in 1-octen-3-ol and 10-oxo-(E)-9-decenoic acid (10-ODA) by a hydroperoxide lyase (Teshima *et al*. 2022). 1-octen-3-ol is transformed to 3-octanone due to the activity of enone reductase in *S. cerevisiae* (Darriet *et al*. 2002). In *Aspergillus* species the gene producing 10-HPOD has been determined as the *ppo*C gene (Brodhun *et al*. 2010; Kataoka *et al*. 2020).

This study successfully identified a substantial portion of BGCs and associated metabolites previously documented within the *Metarhizium* genus, i.e. in *M. anisopliae* (Gao *et al*. 2011; Sbaraini *et al*. 2016), *M. acridum* (Gao *et al*. 2011), and *M. robertsii* (Zhang *et al*. 2020; Sun *et al*. 2022). The number of predicted clusters in both V275 and ARSEF 4556 strains closely resembled that of *M. anisopliae*, totaling 73 clusters (Sbaraini *et al*. 2016). While common BGCs encoding enzymes to produce destruxins, swainsonine, and other well-known secondary metabolites, including products from NRPS and polyketide clusters, were identified for *M. brunneum* (Gao *et al*. 2011; Saud *et al*. 2021), both strains analyzed in this work, exhibited shared and unique clusters not associated with known compounds. These nonassociated BGCs were designated as “cryptic” in this work. The observed variations in entomopathogenic and endophytic activities are postulated to be correlated with different compounds linked to the predicted BGCs (Supplementary Table 10). However, as recommended in a review on BGCs (Keller 2015), further exploration into the transcriptional activity and extent of transcription of these clusters is warranted for a more comprehensive understanding.

Several metabolites such as choline and enniatins were previously acknowledged to exist in *Metarhizium* spp (Zhang *et al*. 2020). However, for the first time, this study established a correlation between these metabolites and specific BGCs or relevant genes in *M. brunneum* (Fig. 9; Supplementary Table 10). This represents a novel contribution to the understanding of the genetic basis underlying the biosynthesis of these metabolites in *M. brunneum*.

While in previous studies, certain known secondary metabolites implicated in endophytism, such as peramine (Berry *et al*. 2019), were confirmed in *M. brunneum*, this study additionally identified clusters utilized in pathways for metabolites novel to *Metarhizium* and previously implicated in the endophytic or mycophilic activity of other fungi, such as ε-poly-L-lysine (Christinaki *et al*. 2024). Moreover, the identification of phytotoxic compounds, such as BAB/BAA compounds (Table 2, Fig. 8), raises intriguing questions about their potential activity while *M. brunneum* resides within the plant host or acts as a rhizosphere colonizer. The in-depth in silico characterization of these compounds opens avenues for further investigation into their specific roles and impacts on the host plant. In addition, a newly characterized class of fungal natural products corresponding to ICS BGC was detected (Table 2). The latter is a commonly found domain in ICS cluster, while the presence of *dit* domain represents the ancestral ICS cluster form. Studies have shown that *dit* ICS is more closely related to bacterial ICSs than to other fungal ICSs and it is speculated that fungal and bacterial *dit* ICSs are remnants of a common ancestor (Lim *et al*. 2018; Nickles *et al*. 2023).

### Intraspecies diversity of TEs

In this study, genomic intraspecies variability was well established, but the mechanisms leading to this genome diversity can only be speculated. TEs may be responsible for the observed variation. TEs are known to be important drivers of genome evolution since they induce genomic alterations associated with insertions, deletions, duplications, or translocations and in extent with gene structure and expression of nearby genes (Finnegan 1989; Mariño-Ramírez *et al*. 2005). The genomic TE distribution of both strains was different in both TE numbers and types, which, in extent, may explain the size augmentation of V275 vs the size of ARSEF 4556. V275 genome is abundant in 8 types of TEs belonging to both Class I RNA transposons and Class II DNA transposons, while ARSEF 4556 appears to have only Class I, with an abundance of TEs belonging to the type LTR/Gypsy as well as RC/Helitron TEs that belong to Class II (Fig. 6). V275 presented a significant number of MULE/MuDR TEs while ARSEF 4556 none (Supplementary Table 8). MULE elements were first identified in maize, but they have been found in several members of animals, protozoans, other plants and fungi (Feschotte and Pritham 2007). Among these, MuDR-MULEs have the highest transposition frequency in maize and a tendency to insert into or near genes (Cresse *et al*. 1995). This result was also found in this work, and it is in accordance with a previous study in which it was shown that the distribution of TEs in fungal genomes can vary significantly among strains of the same species (Daboussi and Capy 2003). Furthermore, this different TE distribution in closely related strains (of the same species) may be attributed to an insertion event and subsequent multiplication through the mechanisms of transposition. The abundance of LTR/Gypsy TEs in the genome of ARSEF 4556 indicates an extensive replication by copy and paste mechanism compared to V275. This idea is further supported by the high number of small-sized MULE/MuDR TEs found in the genome of V275 that may have remained after transposition (Supplementary Table 7 and Supplementary File 1). Furthermore, previous studies have shown that TE distribution may play an important role in the evolution of fungal genomes, since they have been found to cluster in regions with high duplication and recombination events (Thon *et al*. 2006) and are involved in inversion of genomic regions (Braumann *et al*. 2008). Analysis of TEs in this work showed that several TEs of all major types are located next to genes that are exclusively found either in V275, or in *M. brunneum* lineage, suggesting that TEs are major generators of genetic diversity between these strains (Fig. 7; Supplementary Table 8). Most of these lineage-specific genes are associated with pathogenicity or stress responses and do not belong to the typical housekeeping genes, in accordance with previous work (Klosterman *et al*. 2011). The investigation of such genes can provide insights regarding specific traits exhibited by these strains that may allow adaptation to new host niches, as suggested previously for the phytopathogen *V. dahliae* (Klosterman *et al*. 2011). Additionally, these V275 and *M. brunneum* lineage-specific genes may be independent evolutionary incidents as revealed by this analysis. This hypothesis agrees with the one proposed for genus *Metarhizium* which suggests that a great inter and intraspecies genetic variability may be linked to their ability to adapt in various habitats (Bidochka *et al*. 2001). Further research is warranted to evaluate the expression of these strain-specific genes, as well as genes associated with secondary metabolite production, to better understand the mechanisms underlying the observed variations.

### Concluding remarks

Overall, presented herein is the first report of a detailed comparative whole genome analysis of 2 strains of the endophytic entomopathogenic species *M. brunneum* showing a remarkable intraspecies diversity. While in the last few years, advancements have been made in researching the interactions between pathogens and hosts, a significant portion of genes are associated with secondary metabolite production in *Metarhizium* spp. which remains uncharacterized. Additionally, there is limited knowledge about the genomic organization, expression, and regulation of these genes. In detail, content diversity was identified in genes related to secondary metabolism affecting insect pathogenicity mechanisms, endophytism, and antagonism with other microorganisms that also have the same niche. Therefore, this study offers new insights on the genes involved in the adoption of the *M. brunneum* dual mode of life. As previously suggested, HGT events may have played a role in shaping the observed variations in metabolic potential (Khaldi *et al*. 2008; Sbaraini *et al*. 2016) and genomic features found in this work may be related to the differentiation in the endophytic or entomopathogenic abilities of the 2 strains examined here. Thus, an evolution of gene families and mechanisms that are pivotal in modulating response to ecological interactions is unveiled through this study. Furthermore, the existence of several plant pathogenesis-related genes, biosynthetic gene clusters associated with phytotoxic compounds as well as the variety of CAZy enzymes for plant-degrading material highlights the complicated relationships of *Metarhizium* strains, and in extent EPF, with plants. In addition, the investigation of lineage-specific genes is a useful tool to determine putative genetic mechanisms implicated in the differential efficacy of each strain regarding entomopathogenicity and endophytism. This investigation can also shed light on the evolutionary events that have shaped each *Metarhizium* species and their association with both plants and insects.

## Supplementary Material

jkae190_Supplementary_Data

## Data Availability

The genome of V275 has been submitted to the NCBI Genome Databank under BioProject Number PRJNA1057712 and Assembly Accession number GCA_039795395.1. The Supplementary Material for this article can be found online at “figshare” https://doi.org/10.6084/m9.figshare.26151751.v1 Supplemental material available at G3 online.
