# Supplementary material for: Comparative genomics of Metarhizium brunneum strains V275 and ARSEF 4556: unraveling intraspecies diversity
Source: G3 (Bethesda). 2024 Aug 30;14(10):jkae190. doi: 10.1093/g3journal/jkae190 (PMC11457142; doi:10.1093/g3journal/jkae190)
Supplement: jkae190_Supplementary_Data [file jkae190_supplementary_data.docx]

**Supplementary data** can be found online at “figshare” doi: 10.6084/m9.figshare.25447144:

**Table S1:** BUSCO scores of (a) genomes and (b) predicted protein sets of V275 and ARSEF4556 strains.

**Table S2**: The predicted signal domains of V275 proteins.

**Table S3:** Predicted transporter genes in *M. brunneum* V275 genome.

**Table S4:** V275 protein Accession numbers from InterPro, PFAM and MEROPS databases.

**Table S5:** Functional categories based on KEGG and COG annotation**.**

**Table S6:** The families of predicted CAZ enzymes for both strains. Colours indicate variation in the number. Secreted enzymes of V275 are also presented.

**Table S7:** The predicted TEs of genomes V275 and ARSSF4556. Their conserved domain, the class and location, length and sequence are presented.

**Table S8**: The type, ID, size and location of the TEs that contain a Conserved domain. In addition, two genes before and two genes after the predicted TEs are reported, along with their Conserved domain. Red indicates that the gene is solely found in V275 among all *Metarhizium* species, while Green indicates that it can be found within *M. brunneum* lineage.

**Table S9:** The PHI genes found in (a)V275 (b)ARSEF 4556 genomes and (c) their unique PHI genes.

**Table S10:** Clusters of V275 and ARSEF4556 grouped in families and unique clusters located after MIBiG analysis. These clusters are not associated with a known compound.

**Table S11:** The proteins of V275 that are shared between other *Metarhizium brunneum* strains as well as with other *Metarhizium* species. For the search, tblastn against NCBI databases was used. The first two similarity hits along with their identity and coverage are presented. Domain search was performed using CDD database (NCBI). The CDD domain as well as the accession number and hit type are presented.

**Figure S1:** The mitochondrial genome of *M. brunneum* strain V275

**Figure S2**: **The percentages of PHI genes hits associated with each activity.** The percentages in both genomes are the same (e-value 10^5).

**Figure S3**: GO term annotations of genes encoding proteins with conserved domains, found only (a) in all three *Metarhizium* *brunneum* strains ARSEF4556, ARSEF3297 and V275 and (b) only in V275 strain.
